# A Practical Ambulatory Approach to Atrioventricular Block Secondary to Lyme Carditis

**DOI:** 10.19102/icrm.2023.14031

**Published:** 2023-03-15

**Authors:** Carmela Aromin, Anupa Chanda, Sharath Kumar, Garry Robert Thomas

**Affiliations:** ^1^Division of Cardiology, Department of Medicine, Unity Health Toronto (St. Joseph’s Health Centre), Toronto, Ontario, Canada; ^2^Division of Cardiology, Department of Medicine, QEII Health Sciences Center, Halifax, Nova Scotia, Canada; ^3^Faculty of Medicine, University of Toronto, Toronto, Ontario, Canada

**Keywords:** Atrioventricular dissociation, Lyme carditis, temporary permanent pacemaker

## Abstract

Lyme carditis (LC) is a potentially reversible cause of complete atrioventricular (AV) dissociation that rarely requires a permanent pacemaker. The time to resolution is variable, sometimes requiring weeks, making a temporary permanent pacemaker (TPPM) a suitable bridge to recovery. We report on a 31-year-old man with serology-confirmed Lyme disease with complete heart block during the peak of the coronavirus disease 2019 pandemic. A TPPM was implanted and the patient was discharged the following day with regular follow-up in the ambulatory setting. Once 1:1 AV conduction was reestablished, the TPPM was removed. Our case demonstrates that the use of a TPPM for AV-dissociation secondary to LC is a safe and feasible strategy in select individuals which can minimize patient morbidity as well as hospital length of stay and overall health care costs.

Lyme carditis (LC) is a potentially reversible cause of acquired atrioventricular (AV) block, which can present with varying degrees of severity.^1^ Early recognition and treatment with antibiotics and bradycardia support often has a favorable prognosis; however, the duration of pacing required is uncertain and can sometimes be several weeks. As a result, the hospital length of stay is often unpredictable, resulting in increased patient morbidity and health care system challenges in the form of increased costs and reduced accessibility of other patients to hospital beds. We present the use of a temporary permanent pacemaker (TPPM) as a safe and effective strategy for early hospital discharge and ambulatory management of transient complete heart block (CHB) secondary to LC.

## Case presentation

**Figure 1:**
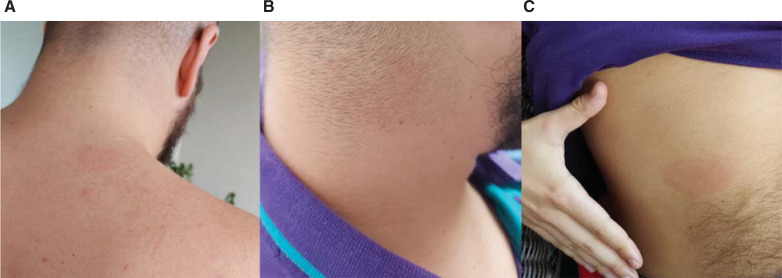
Bullseye rash seen on the back, neck, and trunk of the patient. The patient’s **(A)** back, **(B)** neck, and **(C)** trunk.

**Figure 2:**
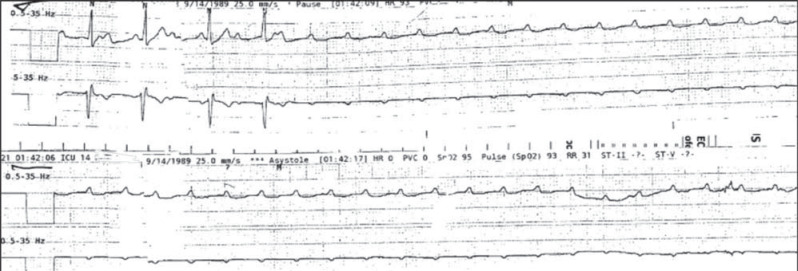
Electrocardiogram at admission showing ventricular asystole for 31 s.

A 31-year-old previously healthy man presented to the hospital with multiple episodes of syncope. One month prior, he had been camping in Ontario, Canada, and found a tick burrowed in his skin. Weeks later, he developed fever, malaise, and multiple rashes suspicious for erythema migrans **(Figures 1A–1C)**. At hospital presentation, his continuous monitoring showed CHB with multiple significant episodes of ventricular asystole **(Figure 2)**. A temporary transvenous pacemaker (TTVP) was inserted via the right internal jugular vein. His white blood cell count and C-reactive protein level were elevated (15.3 × 10^9^/L and 108 mg/L, respectively) with a normal troponin concentration and left ventricular function according to echocardiography. Hilar adenopathy was absent according to chest computed tomography. Applying the established Suspicious Index in Lyme Carditis score, which boasts an impressive sensitivity of 93.2%, the patient received the maximum possible score of 12 points, suggesting a high suspicion for LC.^2^ Consequently, he was treated empirically with 2 g of ceftriaxone intravenously daily, and his serology was confirmed positive 5 days later.

**Figure 3:**
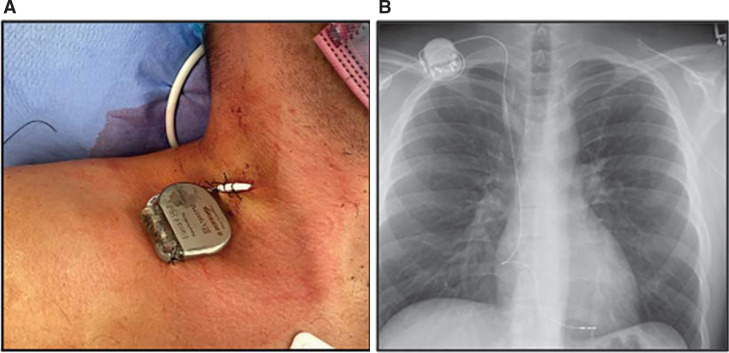
A single-chamber temporary permanent pacemaker. **A:** Implantation via the right internal jugular vein. **B:** Chest X-ray confirming apical placement of the right ventricular lead via the internal jugular vein.

The decision to offer him a permanent pacemaker (PPM) was deferred, recognizing the likely favorable prognosis of this disease. The patient was managed in the coronary care unit. On day 5 of antibiotics, his pacing burden with the TTVP (backup rate, 40 bpm) remained high with only brief and rare evidence of AV conduction. This hospitalization uniquely coincided with the peak of the coronavirus disease 2019 (COVID-19) pandemic locally, when hospital admission rates regularly exceeded capacity, resulting in a systemic emphasis on safe but rapid patient turnover/discharges. A decision was made to implant a TPPM on day 6 of admission **(Figures 3A and 3B)**. An active fixation lead (Solia S 60 cm; Biotronik, Berlin, Germany) was positioned in the right ventricular apex and connected to an externalized single-chamber pulse generator (Biotronik Edora 8 SR-T). The implantation was unremarkable. Rather than transferring the patient to a hospital ward (non-critical care) bed while awaiting recovery from CHB, he was discharged home after 24 h of observation, clearance from physiotherapy, and explicit patient/family education by the cardiology team. An outpatient nurse was assigned to help with the completion of his 21-day course of antibiotics.

**Figure 4:**
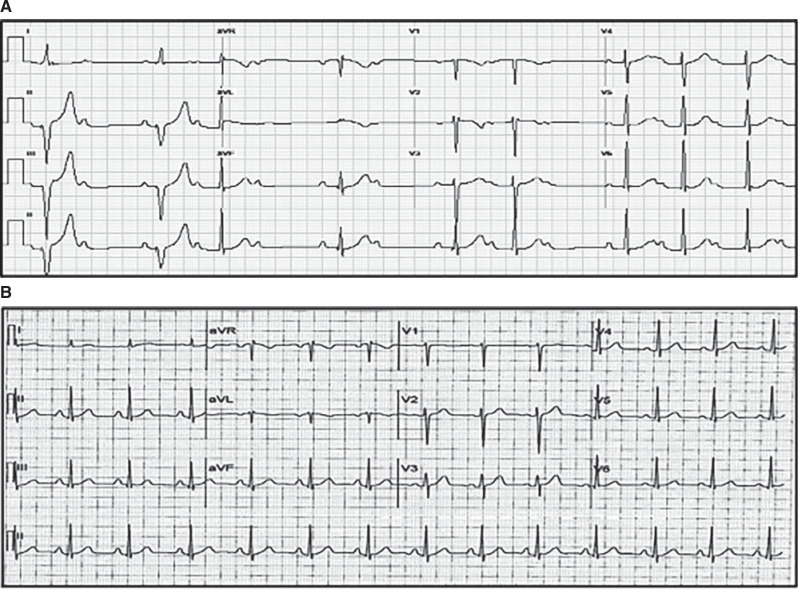
**A:** Postoperative day 3 electrocardiogram showing intermittent ventricular pacing and Wenckebach conduction. **B:** Postoperative day 18 electrocardiogram showing sinus rhythm with 1:1 atrioventricular conduction.

His 12-lead electrocardiogram (ECG) on postoperative day 3 showed Wenckebach conduction **(Figure 4A)** with 14% ventricular pacing on interrogation. Following the completion of his antibiotics (postoperative day 14), a 48-h Holter monitor was arranged, which showed 1:1 AV conduction throughout. Adapting the protocol recommended for hospitalized LC patients with CHB and a TPPM, a 12-lead ECG and a stress test were performed; there was no evidence of ventricular paced beats, high-grade AV block, or Wenckebach conduction, and the patient had a narrow QRS complex with a physiologic P–R interval **(Figure 4B)**.^3^ The TPPM was then interrogated; the pacing diagnostic trend showed a gradual decline to 0%, and this trend had remained constant over the preceding 10 days. The endocardial lead was removed, and the patient was followed up as an outpatient thereafter for 6 months to assess for late complications.

## Discussion

LC occurs in up to 10% of untreated patients with Lyme disease, and 80%–90% of these individuals develop AV dissociation.^3^ The mechanism of cardiac involvement in Lyme disease is related to the direct infiltration of the myocardium and conduction tissue by the spirochete *Borrelia burgdorferi*.^1^ For severe cases of LC that lead to myocarditis or CHB, intravenous ceftriaxone for 14–21 days is the therapy of choice. Complete recovery of AV conduction is usually observed in up to 95% of patients by 4–6 weeks postadmission if they remain compliant with management, resulting in a lower requirement for permanent pacing.^3,4^ In the event of delayed recovery of AV conduction, a TPPM with an active fixation lead is a valuable strategy after TTVP, which allows for better stability and early patient mobilization.^5^ In this report, we highlight the use of a TPPM as a bridge to AV node recovery and its ability to facilitate early and safe discharge from the hospital.

Prolonged hospitalization is expensive. In Canada, the reported daily costs of critical care and ward beds are $3,545 CAD ($2,642 USD) and $2,079 CAD ($1,549 USD), respectively.^6^ By contrast, the cost of an active fixation endocardial lead and single-chamber pulse generator at our center is $1,200 CAD, with the latter’s true cost, as it can theoretically be recycled for future cases, depending on individual hospital policies. At the peak of the COVID-19 pandemic in Canada, the need for hospital beds, particularly in critical care units, was 40% greater than hospital capacity, and the situation in our center was no exception.^7^ This led to an unprecedented need to identify strategies for safe but early patient discharges. Yeung et al. outlined an effective algorithm for the early detection and management of CHB secondary to LC, which minimizes the use of PPMs, but this involves monitoring inpatients, potentially for weeks, until AV conduction has recovered. During the COVID-19 pandemic, adopting this approach was impractical and could have endangered other patients who were denied access to a hospital bed.^3^ Our strategy, in contrast, overcame several of these challenges, but it was not without increased risk and should only be considered in select individuals. TPPMs carry a greater risk of dislodgement than PPMs, and this can result in potentially life-threatening consequences if the individual is pacemaker-dependent and does not remain in a monitored setting. In our case, the patient was highly engaged, as was his family, and was competent to appreciate the risks of hospital discharge prior to the resolution of his CHB. The patient also lived near the hospital with a flexible employer and so there was no perceived barrier to regular follow-up visits. Finally, the nurse assigned to administer daily intravenous antibiotics at the patient’s home was given special instructions regarding the surveillance and management of the pacemaker site. In this setting, and with informed consent, our team felt this would be a safe and appropriate strategy to implement.

## Conclusion

Placement of a TPPM resulting in early hospital discharge can be a practical, safe, and feasible approach to effectively manage CHB as a consequence of LC in select patients. Although conduction disease in this context is often transient, predicting the time to recovery with antibiotics can be challenging. A TPPM can serve as a bridge to recovery, allowing for early mobilization and hospital discharge, reducing health care costs while liberating hospital beds for others in need, and can optimize patient comfort and overall satisfaction.
